# Effect of untreated pharmaceutical plant effluent on cardiac Na^+^-K^+^- ATPase and Ca^2+^-Mg^2+^-ATPase activities in mice (*Mus Musculus*)

**DOI:** 10.1016/j.toxrep.2019.05.002

**Published:** 2019-05-06

**Authors:** A.O. Abdulkareem, T.F. Olafimihan, O.O. Akinbobola, S.A. Busari, L.A. Olatunji

**Affiliations:** a Animal Physiology Unit, Department of Zoology, University of Ilorin, Ilorin, Nigeria; b Ecology and Environmental Biology Unit, Department of Zoology, University of Ilorin, Ilorin, Nigeria; c HOPE Cardiometabic Research Team, and Department of Physiology, College of Health Sciences, University of Ilorin, Ilorin, Nigeria; d HOPE Cardiometabolic Research Team, University of Ilorin, Ilorin, Nigeria

**Keywords:** Effluent, Na^+^-K^+^-ATPase, Ca^2+^-Mg^2+^-ATPase, Cardiac, *Mus musculus*

## Abstract

Cardiovascular diseases are major causes of non-communicable diseases (NCDs)-related throughout the world. Water pollution has been linked with the high global NCD burden but no report exists on the cardiotoxicity of untreated or poorly treated pharmaceutical effluent, despite its indiscriminate discharge into the aquatic environment in Nigeria, as in many other locations of the world. Thus, this study investigated the cardiotoxic effect of oral exposure to pharmaceutical effluent in mice. Thirty (30) male mice (*Mus musculus*) were randomly divided into 6 groups. Group A (control) received 0.2 ml distilled water, while groups B-F were treated with 0.2 ml 2.5%, 5.0%, 10.0%, 20.0% and 40% concentrations (v/v, effluent/distilled water) of the effluent respectively, for 28 days. Significant reductions (*p<*0.05) in heart weight and cardiac weight index were observed in the groups treated with 5%, 10%, 20% and 40% concentrations of the effluent, without significant change in body weight. Similarly, 28 day administration of the effluent showed significant decrease in cardiac Na^+^-K^+^-ATPase activity (*p<*0.05) at concentrations 10% and above, in a concentration dependent manner. However, there was insignificant decrease in cardiac Ca^2+^-Mg^2+^-ATPase activity of the exposed mice, when compared with the control group. This study provides novel information on the cardiotoxic effects of oral exposure to untreated pharmaceutical effluent, showing reduced Na^+^-K^+^-ATPase activity and decreseased myocardial atrophy. Therefore, drinking water contaminated with pharmaceutical effluent may promote the incidence of cardiovascular diseases. Further studies on the exact mechanistic routes of the induced cardiotoxicity are recommended.

## 1 Introduction

Many pharmaceutical companies in Nigeria, as in many other parts of the world, commonly discharge their effluents directly into aquatic environment [1]. These effluents often contain toxic substances as they are either not treated at all or poorly treated [2,3]. Hence, upon release to the environment, they damage aquatic life, constitute environmental threat and probably incorporate into food chains [4,5]. Furthermore, occurrence of pharmaceuticals in both surface and ground waters has been reported [[6], [7], [8], [9]]. Meanwhile, long exposure to trace amount of pharmaceuticals in drinking water could be detrimental to human health [10]. Thus, contamination of drinking water with pharmaceuticals might be a serious public health concern.

Currently, there is an increased incidence of non-communicable diseases (NCDs) in Africa, with high mortality rate [11]. Majority of NCDs related deaths are attributed to cardiovascular diseases (CVDs) alone. CVDs are the leading causes of death worldwide, with over 17.3 million annual deaths [12,13]. This group of diseases remains a major cause of morbidity and mortality in sub-Saharan Africa, with rising incidence and emergence of ischaemic heart disease in Nigeria [14]. Common known risk factors for CVDs are cigarette smoking, elevated cholesterol, hypertension, obesity, physical inactivity and diabetes [15]. However, environmental pollution has been recently suggested as a potential risk factor for cardiorenal metabolic syndrome, including CVDs [16].

Several studies have previously reported the systemic toxicity and genotoxicity of untreated pharmaceutical effluent in both aquatic and terrestrial animals [1,[17], [18], [19], [20], [21]]. These effects, which include anaemia, oxidative stress, histopathological lesions and increased micronucleated polychromatic erythrocytes formation in bone marrow, are presumed to be due to high concentrations of heavy metals such as iron, manganese and other toxic constituents of the effluent [1]. When discharged into water body, heavy metals accumulate in aquatic life, from where they may enter the food chain and cause serious harm to human health [22,23]. However, despite the association of environmental pollution and CVDs, and indiscriminate discharge of untreated or poorly treated pharmaceutical effluents in to aquatic environment in Nigeria, cardiotoxicity of the effluent has not been reported in the region. In this study, we investigated the effect of pharmarceutical effluent on mice cardiac functioning, using Na^+^/K^+^-ATPase and Ca^2+^/Mg^2+^-ATPase assays.

Na^+^/K^+^-ATPase and Ca^2+^/Mg^2+^-ATPase are integral membrane proteins, that mediate transport of ions such as Na^+^, K^+^ and Ca^2+^ across the plasma membrane of almost all animal cells, including myocardium [24,25]. Ion regulation and the maintenance of ion gradients across the cell membrane are important for cell homeostasis, hence, ionic imbalances may result in pathological conditions [26]. More so, control of intracellular Na^+^ plays a vital role in the regulation of cardiac contractility and the treatment of heart failure [27]. Thus, Na^+^-K^+^-ATPase and Ca^2+^-Mg^2+^-ATPase serve as appropriate biomarkers for organ functioning [28].

## 2 Materials and methods

### 2.1 Effluent collection

Raw effluent was collected from a pharmaceutical plant in Ilorin, Kwara State, Nigeria, from the point of discharge into the environment and transported in to the laboratory, Zoology department, University of Ilorin, Ilorin, Nigeria. The collected effluent was filtered; the pH was taken and then stored at 4 °C until it was ready for use.

### 2.2 Physico-chemical properties and heavy metal analysis

Chemical oxygen demand (COD), total dissolved solids (TDS), alkalinity, biochemical oxygen demand (BOD), chlorides, nitrates, ammonia, and phosphates were analyzed, using the methods of APHA (1998). Concentration of metals such as cadmium (Cd), chromium (Cr), iron (Fe), zinc (Zn), nickel (Ni), manganese (Mn), copper (Cu), and lead (Pb) were determined, using standard analytical methods [29,30].

### 2.3 Animals and experimental design

Thirty (30) male mice (*Mus musculus)* of 8–10 weeks old were obtained from Central Research Laboratory, University of Ilorin, Ilorin, Nigeria. They were kept in a clean, frequently disinfected and well ventilated animal house at the Department of Zoology, University of Ilorin and acclimatized for 2 weeks. The animals had unrestricted access to food and water, and were kept in accordance with the regulation of University of Ilorin Ethical committee and in conformity to the NIH Guidelines on the care and use of laboratory animals. The mice were divided into 6 groups. Group A (control) mice received 0.2 ml distilled water, while groups B-F received 0.2 ml 2.5%, 5.0%, 10.0%, 20.0% and 40% concentrations (v/v, effluent/distilled water) of the effluent, respectively. All treatments were administered orally for 28 days.

### 2.4 Tissue preparation

At the end of the experiment, the animals were sacrificed by cervical dislocation. The heart was quickly excised, cleared of connective tissue, weighed and homogenized in ice-cold 0.25 M sucrose solution. The resulting homogenates were diluted appropriately with 0.25 M ice-cold sucrose solution (1:5, w = v) and kept frozen overnight [31]. The cardiac weight index (HW = BW) was calculated as previously reported [32].

### 2.5 Enzymes assays

Na^+^-K^+^-ATPase and Ca^2+^-Mg^2+^-ATPase activities were determined spectrophotometrically, as previously reported [28].

### 2.6 Statistical analysis

Data were analyzed as means ± standard error of mean (SEM). Significance was determined by SPSS version 10.0, using analysis of variance (ANOVA), following Duncan Multiple Post-hoc test. Values at *p* less than 0.05 (*p <* 0.05) were considered significant. Bar chats were generated using Sigma Plot, version 10.0.

## 3 Results

### 3.1 Physicochemical properties of the raw pharmaceutical effluent

The results of analysis of heavy metals and physico-chemical properties of the effluent are shown in Table 1. The pH value (6.40) was within permissible standard of NESREA (6.0–9.0). Concentrations of iron (Fe) and manganese (Mn) and ammonia (NH_3_) in the sample were higher than the national and international allowable limits, while Cadmiun (Cd), Chromium (Cr), Zinc (Zn), and Copper (Cu), phosphate, alkalinity and TDS were lower. Lead (Pb) and nickel (Ni) were below detectable limits.

**Table 1:** Heavy Metals and Physico-Chemical Parameters of the pharmaceutical effluents.

Parameters	Effluent	NESREA^a^	USEPA^b^
pH	6.40	6.0–9.0	6.5–8.5
Nitrate	1.26	10	10
Ammonia	32.50	10	0.02
BOD	16	50	–
COD	62	90	410
Phosphate	2.0	4.62	–
Alkalinity	32.00	150	20
Cd	0.001	9.02	0.005
Cr	0.038	0.05	0.10
Fe	6.85	–	0.30
Zn	0.320-	5.0	
Ni	BDL	0.05	–
Mn	0.10	0.02	0.05
Cu	0.45	0.5	1.0
Pb	BDL		

All values are in mg/L except pH.

BDL - below detectable limit; BOD - biochemical oxygen demand; COD - .

a National Environmental Standards and Regulations Enforcement Agency (2009), Nigeria maximum permissible limits for wastewater discharge.

b United State Environmental Protection Agency(1989).

### 3.2 Effect of chronic exposure to pharmaceutical effluent on body weight and heart weight in mice

Table 2 shows the effect of oral exposure to pharmaceutical effluent on body weight, post-mortem heart weight and cardiac weight index. There was no significant change in the body weight of the mice exposed to the effluent (*p>*0.05). However, the effluent at 5%, 10%, 20% and 40% concentrations significantly reduced the heart weight and cardiac weight index (*p<*0.05).

**Table 2:** Body weight and cardiac mass index of mice exposed to pharmaceutical effluent.

Group	Body weight (g)	Heart Weight (g)	Cardiac mass index (mg/g)
CONTROL	33.1 ± 1.4	0.38 ± 0.04	1.16 ± 0.19
2.5%	32.6 ± 2.8	0.36 ± 0.02	1.15 ± 0.17
5%	37.4 ± 0.6	0.24 ± 0.01*	0.63 ± 0.04*
10%	33.8 ± 2.1	0.07 ± 0.02*	0.22 ± 0.08*
20%	32.7 ± 1.1	0.14 ± 0.01*	0.42 ± 0.01*
40%	36.4 ± 0.8	0.17 ± 0.02*	0.48 ± 0.06*

* *p* < 0.05 vs control across the column.

### 3.3 Effect of chronic exposure to pharmaceutical effluent on cardiac Na^+^-K^+^-ATPase and Ca^2+^-Mg^2+^-ATPase activities in mice

**Fig. 1:**
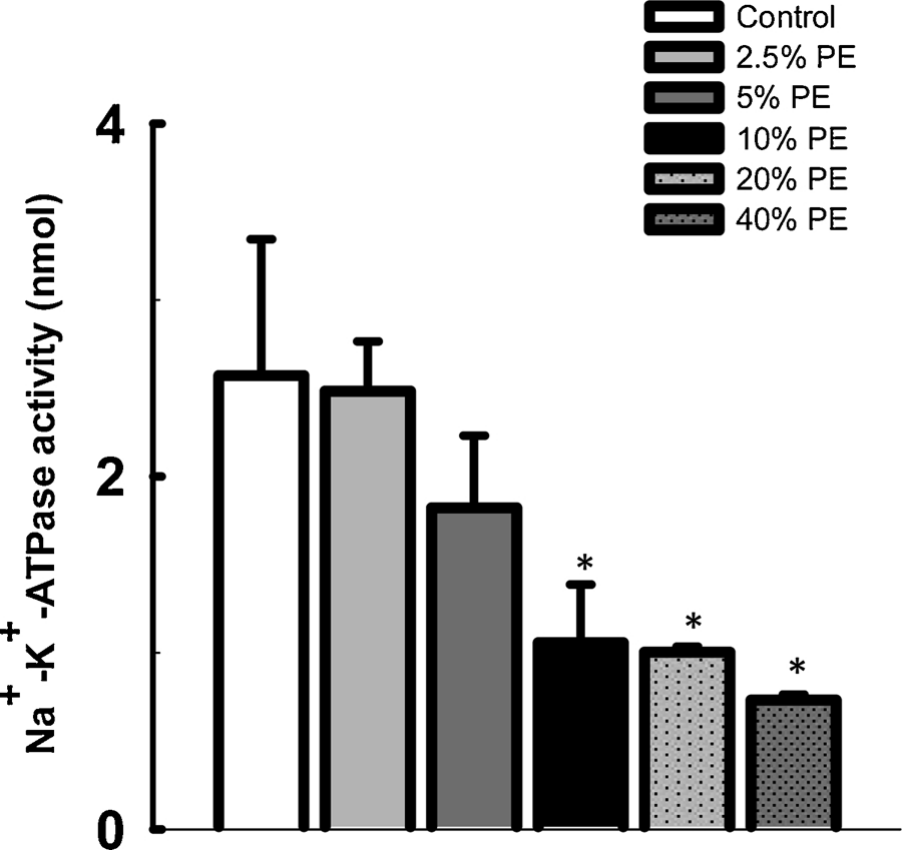
Effect of pharmaceutical effluent (PE) on cardiac Na^+^-K^+^-ATPase activity. (Concentration 10% and above significantly reduced cardiac Na^+^-K^+^-ATPase activity. *p < 0.05 vs control).

**Fig. 2:**
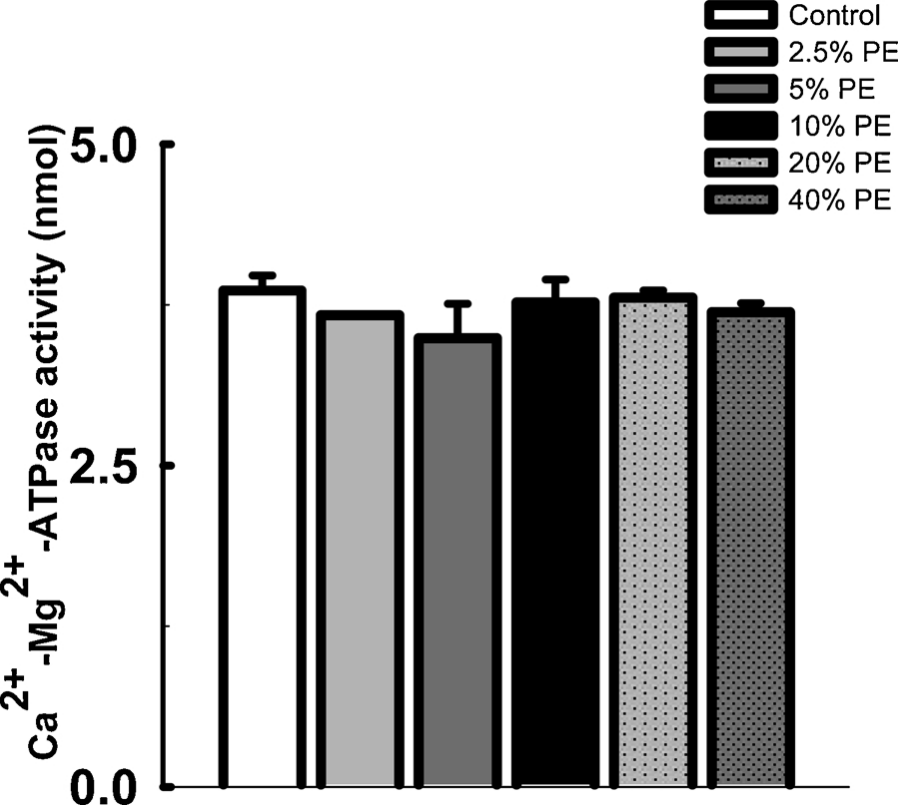
Effect of pharmaceutical effluent (PE) on cardiac Ca^2+^-Mg^2+^-ATPase activity. The effluent slightly reduced cardiac Ca^2+^-Mg^2+^-ATPase activity with no statistical difference.

The results of the effect of pharmaceutical effluent on the cardiac Na^+^-K^+^-ATPase and Ca^2+^-Mg^2+^-ATPase activities in the exposed mice were presented in Fig. 1, Fig. 2, respectively. Chronic administration of the effluent significantly decreased the cardiac Na^+^-K^+^-ATPase activity (*p<*0.05) at concentrations 10% and above, when compared with the control mice (Fig. 1). The effect was concentration dependent, with the least activity observed in those treated with 40% effluent. On the other hand, activity of Ca^2+^-Mg^2+^-ATPase in the heart of exposed mice was not significantly different from the control group (Fig. 2).

## 4 Discussion

The incidence of CVDs is on alarming rate in Africa, including Nigeria. Meanwhile, cardiorenal metabolic syndrome has been previously associated with environmental pollution [18]. In this study, we showed that untreated pharmaceutical plant effluent significantly decreased cardiac Na^+^-K^+^-ATPase activity, heart weight as well as cardiac weight index, and slightly lowered cardiac Ca^2+^-Mg^2+^-ATPase activity in mice. Na^+^-K^+^-ATPase (Na/K pump) functions to regulate active ion transport, maintain electrochemical gradient across the membrane of excitable tissues and control cardiac contractility [33]. On the other hand, Ca^2+^-ATPase increases sequestration of intracellular calcium (Ca), upon its increase, allowing muscle to relax under normal physiological condition [34]. Hence, impairment of Na^+^-K^+^-ATPase as well as Ca^2+^-ATPase may result in ionic imbalance and accumulation of intracellular Ca, which consequently increase the force of heart contraction [35,36]. A number of diseases such as atrial fibrillation [37], ischemia [38,39], heart failure [[40], [41], [42]], hypertension [43,44], atherosclerosis [45] and diabetes [[46], [47], [48]] has been associated with both Na^+^-K^+^-ATPase and Ca^2+^-ATPase dysfunctions. Therefore, decrease in the cardiac activities of these enzymes in the exposed animals, after 28 day oral exposure to pharmaceutical effluent may imply that, chronic exposure to the untreated or partially treated effluent, through drinking water, could induce Ca overload and may result to or promote the incidence of cardiovascular diseases.

Our results showed significant decrease in both absolute heart weight and heart weight index in the exposed mice. Although the reason for this is not clear, it may be due to lysis of cardiac myocytes. Alteration in Na^+^-K^+^-ATPase activity promotes necrosis formation, due to Ca accumulation. Meanwhile, necrosis is characterized by activation of proteolytic enzyme and cell lysis [34,49]. Thus, it can be hypothesized from our results (Table 2) that, oral exposure to untreated pharmaceutical effluent may lead to myocardial atrophy and may be associated with cardiac cachexia. Furthermore, necrosis and reduced cardiac Na^+^-K^+^-ATPase activity, and myocardial atrophy have been linked with ischemia and arteriosclerosis respectively [34,38,50], it is not therefore unlikely that oral exposure to untreated or partially treated pharmaceutical effluent may result to ischemic heart disease and atherosclerosis.

Heavy metals are major contaminants of environmental concern in industrial wastewater [51]. Therefore, cardiotoxic effects of pharmaceutical effluent observed in this study may be attributed to the synergistic effects of its chemical constituents, especially the Mn, Fe and NH_3_ that were above the recommended limits. Both Mn and NH_3_ have been previously reported to lower cardiac activity and muscle contractile function, respectively [52,53]. Similarly, Fe accumulation in the heart increases oxidative stress, leading to cardiomyopathy, muscle atrophy, reduced Na^+^-K^+^-ATPase and Ca^2+^-ATPase activities and heart failure [[54], [55], [56], [57], [58], [59], [60], [61]]. Interestingly, Adeoye and his colleagues [1] have previously reported high lipid peroxidation in rats orally exposed to pharmaceutical plant effluent. This suggests that, the observed cardiac impairment in the exposed mice is in part due to metal-induced oxidative stress.

## 5 Conclusion

Our results showed for the first time, to our knowledge, that, oral exposure to untreated pharmaceutical plant effluent could lead to reduced cardiac weight index and impairment in cardiac Na^+^ -K^+^-ATPase activity. This may imply that drinking water, contaminated with the effluent could increase the chance of developing cardiovascular diseases. Therefore, discouraging indiscriminate discharge of untreated or partially treated pharmaceutical plant effluent into the water body may be helpful in controlling the current global incidence of cardiovascular diseases. Finally, further study investigating the mechanisms of the effluent-induced cardiovascular diseases is recommended.

## Declaration of interests

No financial interest or personal relationship is declared by the authors.

## Author contributions

All the authors have accepted responsibility for the entire content of this submitted manuscript and approved submission.

## Research funding

None declared.

## Employment or leadership

None declared.

## Honorarium

None declared.

## Competing interests

The funding organization(s) played no role in the study design; in the collection, analysis, and interpretation of data; in the writing of the report; or in the decision to submit the report for publication.
